# 

**Published:** 1914-04

**Authors:** 


### CONTENTS.

ORIGINAL COMMUNICATIONS—

The Stomach Tube, by George M. Niles, M. D., Professor of Gastro-Enterology and Clinical Medicine, Atlanta Medical College, Atlanta, Ga. . . . . . . . . . . . . . . . . . . . . 1

A Free Clinic and Does It Pay? by Clarence A. Rhodes, M. D., A. B. . . . . . . . . . . . . . . . . . . . . . . . . . . . . . . . 11

Tracheitis, by R. R. Daly, M. D., Atlanta, Ga. . . . . . . . . . . 15

Fulton County Medical Society, Regular Meeting April 2, 1914. By R. R. Daly, M. D. . . . . . . . . . . . . . . . . . . . . . . 18

EDITORIAL . . . . . . . . . . . . . . . . . . . . . . . . . . . . . . . . . . . . . 22

Selections and Abstracts . . . . . . . . . . . . . . . . . . . . . . . . . . . 23

